# US Hospital Service Availability and New 340B Program Participation

**DOI:** 10.1001/jamahealthforum.2024.0833

**Published:** 2024-05-03

**Authors:** Kelsey M. Owsley, Romana Hasnain-Wynia, Ronica N. Rooks, Gregory J. Tung, Glen P. Mays, Richard C. Lindrooth

**Affiliations:** 1 Department of Health Policy and Management, University of Arkansas for Medical Sciences, Little Rock; 2 Winthrop P. Rockefeller Cancer Institute, University of Arkansas for Medical Sciences, Little Rock; 3 Office of Research, Denver Health and Hospital Authority, Denver, Colorado; 4 Department of Medicine, University of Colorado-Anschutz Medical Campus, Aurora; 5 Department of Health and Behavioral Sciences, University of Colorado Denver, Denver; 6 Department of Health Systems, Management, and Policy, Colorado School of Public Health, University of Colorado-Anschutz Medical Campus, Aurora

## Abstract

**Importance:**

The US 340B Drug Pricing Program enables eligible hospitals to receive substantial discounts on outpatient drugs to improve hospitals’ financial sustainability and maintain access to care for patients who have low income and/or are uninsured. However, it is unclear whether hospitals use program savings to subsidize access as intended.

**Objective:**

To evaluate whether the 340B program is associated with improvements in access to hospital-based services and to test whether the association varies by hospital ownership.

**Design, Setting, and Participants:**

Difference-in-differences and cohort analysis from 2010 to 2019. Never and newly participating 340B general, acute, nonfederal hospitals in the US using data from the American Hospital Association’s Annual Survey of Hospitals merged with hospital and market characteristics. Data were analyzed from January 1, 2023, to January 31, 2024.

**Exposures:**

New enrollment in 340B between 2012 and 2018.

**Main Outcomes and Measures:**

Total number of unprofitable service lines, ie, substance use, psychiatric (inpatient and outpatient), burn clinic, and obstetrics services; and profitable services, ie, cardiac surgery and orthopedic, oncologic, neurologic, and neonatal intensive services.

**Results:**

The study sample comprised a total of 2152 hospitals, 1074 newly participating and 1078 not participating in the 340B program. Participating hospitals were more likely than nonparticipating hospitals to be critical access and teaching hospitals, have higher Medicaid shares, and be located in rural areas and in Medicaid expansion states. At public hospitals, participation in the 340B program was associated with a significant increase in total unprofitable services (0.21; 95% CI, 0.04 to 0.38; *P* = .02) and marginal increases in substance use (5.4 percentage points [pp]; 95% CI, –0.8 pp to 11.6 pp; *P* = .09) and inpatient psychiatric (6.5 pp; 95% CI, –0.7 pp to 13.7 pp; *P* = .09) services. Among nonprofit hospitals, there was no significant association between 340B and service offerings (profitable and unprofitable) except for an increase in oncologic services (2.5 pp; 95% CI, 0.0 pp to 5.0 pp; *P* = .05).

**Conclusions and Relevance:**

The finding of the cohort study indicate that participation in the 340B program was associated with an increase in unprofitable services among newly participating public hospitals. Nonprofit hospitals were largely unaffected. These findings suggest that public hospitals responded to 340B savings by improving patient access, whereas nonprofits did not. This heterogeneous response should be considered when evaluating the eligibility criteria for the 340B program and how it affects social welfare.

## Key Points

**Question:**

Does the US 340B Drug Pricing Program enable participating hospitals to sustain access to hospital-based services and how does hospital ownership affect the influence of 340B participation on hospital service offerings?

**Findings:**

This longitudinal observational study including 2152 general acute care hospitals found that public hospitals were significantly more likely to sustain unprofitable services after 340B participation, but there was not a meaningful association between 340B participation and service offerings at nonprofit hospitals, except for oncologic services.

**Meaning:**

These findings suggest that participation in the 340B program enables public but not nonprofit hospitals to sustain unprofitable service lines, such as psychiatric services.

## Teaser

This cohort study evaluates whether hospital participation in the 340B program was associated with improvements in access to hospital-based services and whether hospital ownership affected service offerings.

## Introduction

The 340B Drug Pricing Program enables participating safety-net hospitals and clinics to receive discounts from pharmaceutical companies on approved outpatient drugs and bill insurers for those drugs at prevailing reimbursement rates. The purpose of the program is to “stretch scarce federal resources as far as possible, reaching more eligible patients and providing more comprehensive services.”^1^ The discount that covered entities receive is quite large, estimated to be around 35%,^2^ and prior research has demonstrated that there is a substantial financial benefit from these discounts.^3^ The number of hospitals in the program has grown substantially during the past 2 decades, from 591 hospitals in 2005 to 1673 in 2011^4^; by 2017, there were 2437 hospitals participating.^5^ By 2021, covered entities spent nearly $44 billion on 340B drugs.^6^ This growth has been accompanied by increased scrutiny of whether covered entities use the financial benefits to improve access as intended.^4,5^

Although there is clear evidence that 340B participation is associated with improved financial performance of covered entities, there is not a consensus as to whether the financial benefits have been used to subsidize care for patients who have low income and/or are uninsured. Several studies have concluded that the 340B program does not spur hospitals to increase care for populations in need of a safety net.^7,8,9,10^ For example, the provision of uncompensated care and community benefits as a whole were largely unchanged at newly participating hospitals.^7,9,10^ There is also evidence that hospitals respond to 340B participation by expanding outpatient services that commonly provide high-cost 340B drugs, such as oncologic services.^8,9,11^

In this study, we extended previous research to evaluate public and nonprofit hospital offerings of relatively unprofitable and profitable services and test whether hospitals respond differently to 340B program participation by ownership or service profitability. We assessed service lines with relatively low reimbursement and high utilization by underinsured patients, such as psychiatric and substance use services, that are especially vulnerable to closure.^12,13,14,15,16^ Public and rural hospitals that serve socially vulnerable communities are more likely to have poor financial performance and be at higher risk of closing unprofitable service lines to remain solvent.^17,18,19^ Communities that encounter service line closures are more likely to experience reduced access to care,^20^ adverse health outcomes,^21,22^ and disruptions in continuity of care,^23^ which may widen disparities for groups of patients living in vulnerable situations. Continued provision of these services reflects a hospital’s mission to provide safety net services, concordant with the goals of the 340B program. We hypothesized that public hospitals would be more likely to use the financial benefits of 340B program participation to support access to care because they are more likely than nonprofit hospitals to tend to serve patients who have low income and are underinsured.

## Methods

This longitudinal cohort study was approved by the University of Arkansas for Medical Science Institutional Review Board and considered exempt because it used only secondary data sources; informed consent was not required for the same reason. We followed the Strengthening the Reporting of Observational Studies in Epidemiology (STROBE) reporting guideline for cohort studies.

### Data Sources

This study used data from the American Hospital Association (AHA) Survey from 2010 to 2019. Hospital service availability was identified using AHA data. The Health Resources and Services Administration Office of Pharmacy Affairs Information System 340B covered entity daily report was used to identify 340B participating hospitals and participation dates. The Centers for Medicare & Medicaid Services Hospital Cost Report Information System and Provider of Services files were used to obtain additional hospital characteristics. We obtained market-level characteristics from the Agency for Healthcare Research and Quality Social Determinants of Health Database. This dataset compiles demographic, socioeconomic, and health variables from several sources. We used variables from the American Community Survey and the US Centers for Disease Control and Prevention (CDC) Wide-Ranging Online Data for Epidemiological Research and the Social Vulnerability Index (SVI). Demographic information including race and ethnicity was self-reported.

### Study Sample

The sample included nonprofit and public (state and locally owned) short-term, general, and acute care US hospitals. Most public and nonprofit hospitals are eligible for the 340B program if they maintain a Disproportionate Share Hospital percentage higher than 11.75%.^24^ For-profit hospitals are not eligible for the 340B program; therefore, they were excluded from the study. The study sample included 2770 short-term general hospitals not participating in the 340B program before 2012. We restricted the study sample to nonparticipating short-term general hospitals to compare newly treated with never treated hospitals, excluding the always treated hospitals, which is an inappropriate comparison group (always and newly treated participant characteristics are presented in eTable 1 in Supplement 1).^25^ We excluded 108 hospitals that were missing AHA data on service availability and 181 hospitals that terminated 340B enrollment during the study period. Lastly, we required 340B participating hospitals to report data for at least 1 year before and 1 year after 340B enrollment; thus, hospitals that enrolled in 2019 were excluded from the sample (329 hospitals excluded). The final sample included 2152 hospitals, of which 1074 hospitals began participation from 2012 to 2018, and 1078 hospitals never participated in the 340B program during the study period.

### Outcome Measures

We used the approach by Horwitz and Nichols^26,27^ to identify profitable and unprofitable clinical services. We included substance use, burn clinics, inpatient psychiatric, outpatient psychiatric, and obstetric services as unprofitable service lines, and cardiac surgery and orthopedic, oncologic, neurologic, and neonatal intensive services as profitable services (AHA definitions are described in the eMethods in Supplement 1). The outcomes included a count of unprofitable and profitable services from 0 to 5, dichotomous indicators for whether any unprofitable or profitable services were provided, and individual service line variables that equal 1 if the hospital directly provided the service. Following the Horwitz approach, we imputed missing service lines if the adjacent observation years were concordant. To confirm that our findings were not due to erroneous reporting in the AHA data, we required hospitals to report at least 2 consistent observation years after a change in service line availability.

Hospital ownership was identified as nonprofit, for-profit, or public, as reported in AHA and validated using Provider of Services files. Other hospital characteristics included the number of patient admissions (<1000, 1000-9999, ≥10 000); hospitals with membership in the Council of Teaching Hospitals; critical access hospital designation; multihospital system status; top quartile of Medicare and Medicaid share, based on the sample distribution; and case-mix index, a weighted measure of admissions by diagnosis-related group reflecting the relative complexity of conditions treated at each hospital.

Hospital market concentration, defined by the Herfindahl-Hirschman Index (HHI) within a Dartmouth Atlas of Health Care Hospital Referral Region (HRR), was included to account for variation in hospital competition. We included for-profit market share, measured by the percentage of discharges from for-profit hospitals in an HRR, because hospital service offerings is dependent on market mix.^26^ For individual service analyses, we included a control for whether the respective service was offered by a competing hospital in the HRR. Analyses also controlled for county-level demographic and socioeconomic variables, including median household income and percentage of patients who were uninsured, of White race, and age 65 years and older (self-reported data from the American Community Survey). We controlled for the county drug death rate by using the CDC Wide-Ranging Online Data for Epidemiological Research data to adjust for demand of substance use services. A time-varying indicator for whether the state participated in the Affordable Care Act’s Medicaid expansion was included using information from the Kaiser Family Foundation.^28^ Rural hospital was defined as being in a county with a Rural-Urban Area Commuting Code of 4 to 9.^29^ We used the SVI to identify hospitals located in socially vulnerable communities.^30^ SVI is a composite measure based on 15 social factors (eg, socioeconomic, racial and ethnic composition, housing and transportation accessibility) ranging from 0 to 100 with higher scores indicating greater community vulnerability (details provided in the eMethods in Supplement 1). We categorized the SVI variable by terciles.

### Statistical Analysis

First, we descriptively compared characteristics between new 340B participants and never participating hospitals by hospital ownership. Bivariate analyses used Student *t* tests for continuous variables and χ^2^ tests for binary variables. Then we estimated difference-in-differences specifications to evaluate how the 340B program affected hospital service line provision. We followed the approach of Callaway and Sant’Anna^31^ to account for variation in treatment timing and test the validity of the pretrends. This approach estimated the treatment effect between each treatment cohort (ie, hospitals that began participating at different years) and year, and aggregated these estimates to obtain an average treatment-effect estimate. We also estimated an event study specification within the Callaway and Sant’Anna framework to assess trends before and after 340B participation.^32^ Primary analyses evaluated all noncritical access hospitals and were stratified by hospital ownership to assess whether public and nonprofit hospitals responded differently to 340B savings. We limited primary analyses to noncritical access hospitals because they typically provide few services and have limited financial flexibility to add services. In subanalyses, we assessed other hospital subgroups that are typically associated with hospital financial performance and community vulnerability. We stratified analyses by geographic location (rural vs urban), community SVI tercile, and critical access designation.

All analyses were based on a linear specification with hospital and calendar year fixed effects. We controlled for hospital- and market-level characteristics hypothesized to be associated with service availability listed in Table 1. When analyzing all hospitals, we included interaction terms between ownership and rurality, and ownership and SVI categories. The analysis sample included up to 5 years of data before and after 340B participation. Standard errors were clustered at the hospital level. Sensitivity analyses used a balanced panel limited to hospitals that reported in every sample year and logistic regression for dichotomous outcome variables (eTables 2 and 3 in Supplement 1). We also assessed whether hospitals added or dropped a service line, by stratifying the sample by whether the service was offered at the start of the study period (eTable 4 in Supplement 1). All tests were 2-sided, and statistical significance was defined as *P* < .05. Analyses were conducted using Stata, version18.0 (StataCorp LLC) from January 1, 2023, to January 31, 2024.

**Table 1.** Baseline Hospital and Market Characteristics for Hospitals Never Participating in the 340B Program and Newly Participating Hospitals in 2012 to 2018, by Hospital Ownership^a^

Characteristic	No. (%)					
	Nonprofit hospitals			Public hospitals		
	Never-340B hospitals	New 340B hospitals	*P* value	Never-340B hospitals	New 340B hospitals	*P* value
Hospitals, No.	920	762	NA	158	312	NA
**Hospital**						
Admissions						
<1000	99 (10.8)	198 (26.0)	<.001	61 (38.6)	185 (59.3)	<.001
1000-9999	502 (54.6)	300 (39.4)	NA	75 (47.5)	85 (27.2)	NA
≥10 000	319 (34.7)	264 (34.6)	NA	22 (13.9)	42 (13.5)	NA
Critical access designation	43 (4.7)	252 (33.1)	<.001	28 (17.7)	199 (63.8)	<.001
Multihospital system	728 (79.1)	540 (70.9)	<.001	63 (39.9)	108 (34.6)	.26
Teaching hospital	29 (3.2)	72 (9.4)	<.001	2 (1.3)	25 (8.0)	.003
Top-quartile Medicaid share	169 (18.4)	343 (45.0)	<.001	59 (37.3)	122 (39.1)	.71
Top-quartile Medicare share	147 (16.0)	187 (24.5)	<.001	67 (42.4)	155 (49.7)	.14
Case-mix index, mean (SD)	1.431 (0.253)	1.483 (0.214)	<.001	1.253 (0.265)	1.457 (0.208)	<.001
**Market**						
Herfindahl-Hirschman Index**, **mean (SD)	0.15 (0.12)	0.19 (0.15)	<.001	0.16 (0.12)	0.18 (0.14)	.08
For-profit market share, mean (SD)	0.10 (0.14)	0.09 (0.12)	.01	0.16 (0.15)	0.16 (0.16)	.92
Social Vulnerability Index, tercile						
Bottom	329 (35.8)	203 (26.6)	<.001	45 (28.5)	90 (28.8)	.81
Middle	323 (35.1)	254 (33.3)	NA	50 (31.6)	90 (28.8)	NA
Top	268 (29.1)	305 (40.0)	NA	63 (39.9)	132 (42.3)	NA
Rural geographic area	214 (23.3)	389 (51.0)	<.001	98 (62.0)	237 (76.0)	.002
State-expanded Medicaid	604 (65.7)	542 (71.1)	.02	55 (34.8)	166 (53.2)	<.001
**County sociodemographic information, mean (SD)**						
Median income^b^	55.0 (14.9)	46.4 (10.5)	<.001	44.0 (11.2)	44.3 (9.7)	.76
% Uninsured	15.4 (5.7)	16.6 (5.5)	<.001	19.1 (5.7)	19.4 (5.6)	.62
% White	78.7 (15.1)	80.8 (17.1)	.008	78.0 (19.4)	82.6 (16.5)	.007
% Age ≥65 y	13.5 (3.6)	14.8 (3.9)	<.001	14.9 (4.0)	15.7 (4.6)	.06
Drug death rate	13.5 (6.1)	15.0 (6.7)	<.001	15.4 (7.2)	16.2 (6.9)	.20

Abbreviation: NA, not applicable.

^a^ Hospitals’ first observation year in the dataset was used. The 340B column represents hospitals that began participating in the 340B program from 2012 to 2018. The comparison hospitals represent those that never participated in the program throughout the study period. The *P* value indicates the difference between new participating hospitals and never 340B hospitals using Student *t* tests for continuous and χ^2^ tests for binary variables.

^b^ Median annual household income is standardized by $1000.

## Results

Table 1 presents sample characteristics by hospital ownership during the first observation year of the study period. Nonprofit hospitals that newly participated in 340B were more likely to have fewer than 1000 admissions (198 [26.0%] vs 99 [10.8%]); be designated as a critical access (252 [33.1%] vs 43 [4.7%]) or teaching hospital (72 [9.4%] vs 29 [3.2%]); be in the top quartile of Medicaid (343 [45.0%] vs 169 [18.4%]) and Medicare (187 [24.5%] vs 147 [16.0%]) share of admissions; and have a higher case mix index (all *P* < .001) than nonparticipating nonprofit hospitals. Participating nonprofits were also less likely to be owned by a multihospital system (540 [70.9%] vs 728 [79.1%]). Similar patterns were found for public hospitals.

Newly participating nonprofits were located in less competitive markets (HHI, 0.19 vs 0.15; *P* < .001) with lower for-profit market shares (0.09 vs 0.10; *P* = .01). Participating nonprofits were generally located in more socially vulnerable areas (305 [40.0%] vs 268 [29.1%]) with lower median household income ($46 400 vs $55 000) and higher uninsured rates (16.6% vs 15.4%; all *P* < .001). Participating nonprofit hospitals were in communities with higher percentages of non-Hispanic White (80.8% vs 78.7%; *P* = .008) and age 65 and older (14.8% vs 13.5%*; P < *.001) populations. New 340B hospitals were also more likely to be in rural areas (389 [51.0%] vs 214 [23.3%]; *P* < .001) and in states that expanded Medicaid compared with nonparticipating nonprofit hospitals (542 [71.1%] vs 604 [65.7%]; *P* = .02). Among public hospitals, new 340B hospitals were more likely to be rural areas (237 [76.0%] vs 98 [62.0%]; *P* = .002) and in expansion states (166 [53.2%] vs 55 [34.8%]; *P* < .001). They also served communities with higher percentage of non-Hispanic White populations (82.6% vs 78.0%; *P* = .007).

Table 2 displays the mean service offerings before and after 340B participation and the unadjusted difference-in-differences estimates. On average, 340B participants offered 1.99 and 1.85 unprofitable services at nonprofit and public hospitals, respectively, before 340B participation. Program participants generally increased their number of unprofitable services, while control hospitals’ services declined. The unadjusted difference-in-differences estimate indicated new 340B hospitals increased total unprofitable services on average by 0.20 (95% CI, 0.11-0.30) and 0.32 (95% CI, 0.07-0.56) at nonprofit and public hospitals, respectively. Program participation was associated with an increase in total profitable services at nonprofit hospitals (0.14; 95% CI, 0.02-0.26), but not public hospitals.

**Table 2.** Unadjusted Service Provisions Before and After New Hospital 340B Program Participation From 2012 to 2018 Compared With Never Participating Hospitals

Hospital ownership and services	New 340B hospitals			Never-340B hospital			Unadjusted D-in-D^d^ estimate (95% CI)^e^	*P* value
	Before^a^	After^a^	Difference^c^	Before^b^	After^b^	Difference^c^		
**Nonprofit hospitals (n = 1387)**								
Unprofitable service lines, mean								
Any unprofitable services	0.961	0.967	0.006	0.854	0.805	–0.049	0.054 (0.025 to 0.084)	<.001
Total unprofitable services	1.990	2.060	0.070	1.509	1.376	–0.133	0.203 (0.108 to 0.298)	<.001
Substance use	0.121	0.126	0.004	0.085	0.060	–0.025	0.029 (0 to 0.058)	.05
Inpatient psychiatric	0.519	0.540	0.021	0.353	0.318	–0.035	0.056 (0.014 to 0.097)	.009
Outpatient psychiatric	0.412	0.444	0.032	0.290	0.264	–0.026	0.058 (0.011 to 0.105)	.02
Burn care	0.047	0.048	0.001	0.013	0.008	–0.004	0.005 (–0.011 to 0.021)	.51
Obstetrics	0.890	0.903	0.013	0.770	0.727	–0.043	0.056 (0.022 to 0.089)	.001
Profitable service lines, mean								
Any profitable services	0.969	0.977	0.008	0.955	0.953	–0.002	0.010 (–0.011 to 0.032)	.36
Total profitable services	3.374	3.535	0.160	3.096	3.115	0.020	0.140 (0.020 to 0.261)	.02
Cardiac surgery	0.444	0.475	0.031	0.377	0.376	–0.001	0.032 (–0.008 to 0.072)	.11
Orthopedic	0.953	0.956	0.003	0.930	0.936	0.006	–0.003 (–0.030 to 0.023)	.81
Oncologic	0.834	0.863	0.028	0.774	0.748	–0.025	0.054 (0.013 to 0.094)	.009
Neurologic	0.775	0.800	0.026	0.756	0.783	0.027	–0.002 (–0.044 to 0.041)	.94
Neonatal intensive	0.373	0.444	0.071	0.260	0.275	0.015	0.056 (0.015 to 0.096)	.008
**Public hospitals (n = 243)**								
Unprofitable service lines, mean								
Any unprofitable services	0.932	0.975	0.043	0.676	0.727	0.051	–0.008 (–0.102 to 0.087)	.88
Total unprofitable services	1.853	2.239	0.386	1.146	1.217	0.071	0.315 (0.066 to 0.564)	.01
Substance use	0.058	0.147	0.089	0.059	0.056	–0.003	0.093 (0.026 to 0.160)	.007
Inpatient psychiatric	0.446	0.579	0.132	0.284	0.275	–0.009	0.142 (0.031 to 0.252)	.01
Outpatient psychiatric	0.365	0.429	0.064	0.183	0.275	0.092	–0.028 (–0.145 to 0.088)	.63
Burn care	0.110	0.170	0.059	0.027	0.012	–0.015	0.074 (0.020 to 0.129)	.008
Obstetrics	0.874	0.915	0.041	0.594	0.602	0.009	0.032 (–0.065 to 0.130)	.52
Profitable service lines, mean								
Any profitable services	0.858	0.890	0.032	0.717	0.745	0.028	0.004 (–0.098 to 0.105)	.95
Total profitable services	2.756	3.055	0.299	1.854	2.006	0.152	0.147 (–0.201 to 0.495)	.41
Cardiac surgery	0.304	0.345	0.041	0.155	0.149	–0.006	0.047 (–0.044 to 0.138)	.31
Orthopedic	0.850	0.882	0.033	0.689	0.714	0.025	0.008 (–0.095 to 0.111)	.88
Oncologic	0.667	0.702	0.036	0.507	0.547	0.040	–0.004 (–0.122 to 0.115)	.95
Neurologic	0.611	0.693	0.083	0.379	0.460	0.081	0.002 (–0.108 to 0.113)	.97
Neonatal intensive	0.332	0.441	0.110	0.123	0.138	0.014	0.096 (–0.002 to 0.193)	.06

Abbreviation: D-in-D, difference-in-differences.

^a^ Before and after periods refer to years before and after 340B participation for new participants. The year of 340B participation is excluded as a washout period.

^b^ For never-340B participants, the before and after periods represent years 2010 to 2011 and 2018 to 2019, respectively.

^c^ Difference column refers to the before and after period differences.

^d^ Unadjusted difference-in-differences estimate refers to the differential change in service offerings in the after period relative to the before period treatment-control difference.

^e^ 95% CIs were calculated using standard errors clustered at the hospital level.

The statistics represent the proportion of hospitals offering the service, except for the total number of profitable or unprofitable services which represent the mean. The sample comprises noncritical access hospitals.

Adjusted difference-in-differences analyses estimating the association between 340B participation and service offerings are presented in Table 3. Across all noncritical access hospitals, the 340B program was not associated with unprofitable service offerings, except for a slight increase in obstetrics services by 1.6 (95% CI, 0.1-3.2; *P* = .04) percentage points (pp). Among nonprofit hospitals, there was no statistically significant change in total or individual unprofitable services. Among public hospitals, participating in the 340B program was associated with a significant increase in the number of unprofitable services on average by 0.21 (or an 11.4% increase relative to baseline) compared with public hospitals that did not participate in the program (95% CI, 0.04-0.38; *P* = .02). Public hospitals were also associated with a marginally significant increase in substance use and inpatient psychiatric services (*P* < .10). Among nonprofit hospitals, the 340B program was significantly associated with an increase in oncologic services by 2.5 (95% CI, 0.0-5.0; *P* = .05) pp, while public hospitals’ profitable services were unaffected. The results were overall robust to sensitivity analyses using a balanced panel and logistic regression (eTables 3 and 4 in Supplement 1).

**Table 3.** Difference-in-Differences Estimates of Service Provisions for Hospitals Newly Participating in the 340B Program Compared With Never Participating, by Hospital Ownership

Services	Difference-in-differences estimate (95% CI); *P* value^a^^,^^b^		
	Overall	By hospital ownership	
		Nonprofit	Public
Hospitals, No.	1630	1387	243
Total unprofitable services	0.032 (–0.024 to 0.088; *P* = .27	–0.001 (–0.058 to 0.057); *P* = .98	0.212 (0.040 to 0.384); *P* = .02
Any unprofitable services	0.004 (–0.015 to 0.024); *P* = .66	0.006 (–0.012 to 0.024); *P* = .54	–0.019 (–0.078 to 0.039); *P* = .52
Substance use	0.010 (–0.011 to 0.032); *P* = .35	0.001 (–0.022 to 0.023); *P* = .96	0.054 (–0.008 to 0.116); *P* = .09
Inpatient psychiatric	0.001 (–0.022 to 0.023); *P* = .93	–0.017 (–0.039 to 0.006); *P* = .16	0.065 (–0.007 to 0.137); *P* = .08
Outpatient psychiatric	0.012 (–0.019 to 0.044); *P* = .44	0.012 (–0.021 to 0.045); *P* = .48	0.060 (–0.035 to 0.156); *P* = .22
Burn care	–0.004 (–0.018 to 0.009); *P* = .52	–0.009 (–0.023 to 0.006); *P* = .24	0.027 (–0.018 to 0.072); *P* = .24
Obstetrics	0.016 (0.001 to 0.032); *P* = .04	0.014 (–0.003 to 0.031); *P* = .10	0.028 (–0.011 to 0.067); *P* = .16
Total profitable services	0.010 (–0.041 to 0.060); *P* = .71	0.022 (–0.032 to 0.077); *P* = .42	–0.094 (–0.279 to 0.092); *P* = .32
Any profitable services	–0.001 (–0.013 to 0.011); *P* = .89	0.008 (–0.001 to 0.018); *P* = .08	–0.061 (–0.136 to 0.014); *P* = .11
Cardiac surgery	–0.013 (–0.027 to 0.002); *P* = .08	–0.014 (–0.030 to 0.002); *P* = .08	–0.011 (–0.057 to 0.034); *P* = .62
Orthopedic	0.001 (–0.015 to 0.018); *P* = .87	0.009 (–0.009 to 0.027); *P* = .32	–0.032 (–0.104 to 0.041); *P* = .39
Oncologic	0.015 (–0.008 to 0.038); *P* = .19	0.025 (0 to 0.050); *P* = .05	–0.020 (–0.093 to 0.052); *P* = .59
Neurologic	–0.001 (–0.024 to 0.022); *P* = .94	0.001 (–0.026 to 0.028); *P* = .93	–0.011 (–0.075 to 0.053); *P* = .74
Neonatal intensive	0.004 (–0.016 to 0.023); *P* = .72	0 (–0.019 to 0.020); *P* = .97	–0.038 (–0.168 to 0.092); *P* = .57

^a^ Displays the coefficient from the difference-in-differences estimate using ordinary least squares regression adjusted for control variables in Table 1 and with hospital and calendar year fixed effects. Results account for staggered entry into the 340B program. The sample comprises non-critical access hospitals.

^b^ 95% CIs are calculated using standard errors clustered at the hospital level.

The Figure reports the event study analysis assessing the trends in the number of services before and after 340B participation among public and nonprofit hospitals. The coefficient represents the difference between participating and nonparticipating hospitals in each year relative to 340B participation initiation (ie, trend year). Public hospitals had slightly fewer unprofitable services relative to nonparticipating hospitals before participation, although the difference was not statistically significant. The preparallel trends assumption was confirmed for both public and nonprofit hospitals. Among public hospitals, the number of unprofitable service lines steadily increased for new 340B participants compared with nonparticipating hospitals in the before and after period, although the increase was steeper in the period after. After participating in the 340B program for 3 years, public hospitals increased the number of unprofitable services on average by 0.32 (95% CI, 0.06-0.57; *P* = .02). There was no change in services at nonprofit hospitals.

**Figure.**
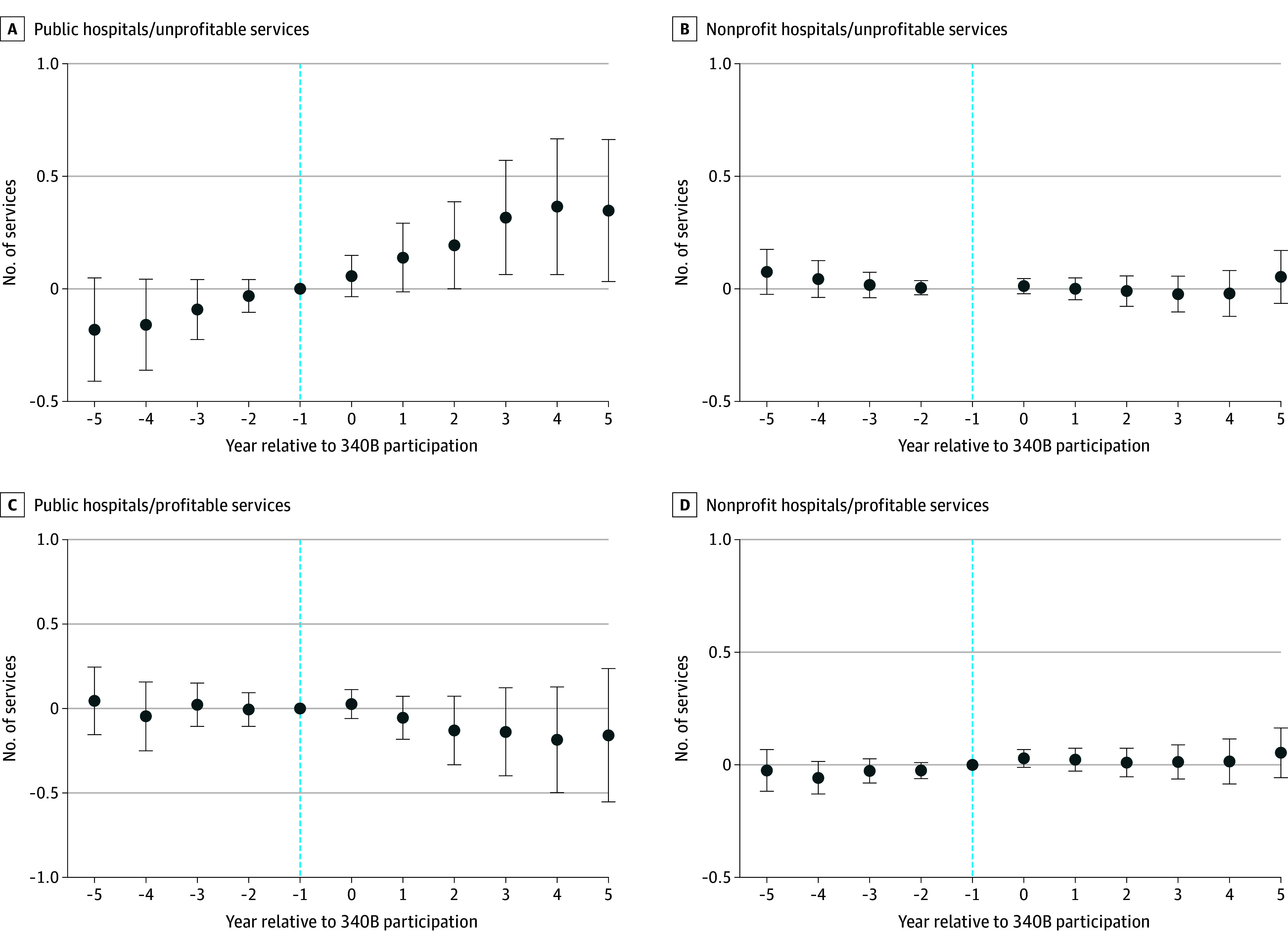
Event-Study Difference-in-Differences of Total Unprofitable and Profitable Services Between Hospitals Participating and Not Participating in the 340B Program, by Hospital Ownership Time at −1, the reference period, indicates the year before 340B participation. Event-study models used adjusted linear regression with hospital and year fixed effects, and standard errors clustered at the hospital level. The sample comprises noncritical access hospitals.

Table 4 displays subanalyses examining all hospitals by rurality, community SVI, and critical access designation. Critical access hospitals slightly decreased burn care services by 1.9 (95% CI, –3.5 to –0.2; *P* = .02) pp. The total number of profitable services increased for hospitals located in the bottom SVI tercile (least socially vulnerable communities). After 340B participation, oncologic services increased at both rural and critical access hospitals by approximately 4 (rural, 95% CI, 1.6 to 7.8; *P* = .003; critical access, 95% CI, 0.7 to 7.3; *P* = .02) pp. Critical access hospitals also significantly decreased neonatal intensive services by 2.3 (95% CI, –3.9 to –0.6; *P* = .007) pp. Noncritical access hospitals slightly increased obstetrics services by 1.6 (95% CI, 0.1 to 3.2; *P* = .04) pp.

**Table 4.** Difference-in-Differences Estimates of Service Provisions for Hospitals Newly Participating in the 340B Program Compared With Never Participating Hospitals, by Rurality, Community Social Vulnerability Index, and Critical Access Designation

Service	Difference-in-differences estimate (95% CI)^a^^,^^b^						
	Geographic location		Community Social Vulnerability Index,^c^ tercile			Critical access hospital	
	Rural	Urban	Bottom	Middle	Top	Yes	No
Hospitals, No.	938	1214	588	672	861	522	1630
Total unprofitable services	0.035 (–0.036 to 0.107); *P* = .33	0.028 (–0.040 to 0.097); *P* = .42	0.051 (–0.074 to 0.176); *P* = .43	0.008 (–0.061 to 0.077); *P* = .82	0.060 (–0.030 to 0.150); *P* = .19	–0.044 (–0.137 to 0.048); *P* = .34	0.032 (–0.024 to 0.088); *P* = .27
Any unprofitable services	0.041 (–0.008 to 0.091); *P* = .10	–0.010 (–0.033 to 0.014); *P* = .41	0.033 (–0.064 to 0.130); *P* = .50	–0.008 (–0.035 to 0.019); *P* = .57	0.045 (0.002 to 0.087); *P* = .04	–0.016 (–0.098 to 0.066); *P* = .71	0.004 (–0.015 to 0.024); *P* = .66
Substance use	–0.005 (–0.020 to 0.009); *P* = .48	0.016 (–0.012 to 0.044); *P* = .27	0.017 (–0.009 to 0.044); *P* = .20	0.007 (–0.019 to 0.032); *P* = .62	–0.002 (–0.031 to 0.027); *P* = .88	–0.012 (–0.027 to 0.002); *P* = .10	0.010 (–0.011 to 0.032); *P* = .35
Inpatient psychiatric	0.006 (–0.024 to 0.035); *P* = .71	–0.003 (–0.028 to 0.022); *P* = .82	–0.008 (–0.033 to 0.018); *P* = .56	–0.010 (–0.040 to 0.019); *P* = .49	0.017 (–0.018 to 0.052); *P* = .34	–0.030 (–0.066 to 0.005); *P* = .09	0.001 (–0.022 to 0.023); *P* = .93
Outpatient psychiatric	0.025 (–0.014 to 0.065); *P* = .21	0.012 (–0.028 to 0.053); *P* = .56	0.037 (–0.043 to 0.118); *P* = .36	0.004 (–0.038 to 0.046); *P* = .84	0.038 (–0.008 to 0.084); *P* = .11	–0.016 (–0.074 to 0.043); *P* = .60	0.012 (–0.019 to 0.044); *P* = .44
Burn care	–0.004 (–0.014 to 0.005); *P* = .37	–0.006 (–0.024 to 0.012); *P* = .54	–0.007 (–0.025 to 0.010); *P* = .40	–0.002 (–0.012 to 0.008); *P* = .73	0.002 (–0.020 to 0.024); *P* = .86	–0.019 (–0.035 to –0.002); *P* = .02	–0.004 (–0.018 to 0.009); *P* = .52
Obstetrics	0.014 (–0.020 to 0.048); *P* = .42	0.011 (–0.007 to 0.028); *P* = .23	0.013 (–0.066 to 0.093); *P* = .74	0.010 (–0.016 to 0.035); *P* = .46	0.009 (–0.012 to 0.031); *P* = .40	0.018 (–0.043 to 0.079); *P* = .56	0.016 (0.001 to 0.032); *P* = .04
Total profitable services	0.072 (–0.006 to 0.150); *P* = .07	–0.033 (–0.089 to 0.023); *P* = .25	0.124 (0.018 to 0.231); *P* = .02	0.046 (–0.037 to 0.129); *P* = .28	–0.036 (–0.104 to 0.032); *P* = .30	–0.012 (–0.102 to 0.078); *P* = .79	0.010 (–0.041 to 0.060); *P* = .71
Any profitable services	0.008 (–0.037 to 0.053); *P* = .74	–0.008 (–0.025 to 0.008); *P* = .31	0.036 (0.001 to 0.071); *P* = .04	0.001 (–0.037 to 0.040); *P* = .95	–0.023 (–0.067 to 0.021); *P* = .30	–0.042 (–0.097 to 0.014); *P* = .14	–0.001 (–0.013 to 0.011); *P* = .89
Cardiac surgery	0 (–0.013 to 0.012); *P* = .96	–0.014 (–0.029 to 0.002); *P* = .08	–0.001 (–0.017 to 0.015); *P* = .91	–0.010 (–0.026 to 0.005); *P* = .20	–0.012 (–0.032 to 0.008); *P* = .25	0.011 (–0.004 to 0.027); *P* = .16	–0.013 (–0.027 to 0.002); *P* = .08
Orthopedic	0.013 (–0.034 to 0.061); *P* = .58	–0.009 (–0.027 to 0.010); *P* = .35	0.060 (–0.002 to 0.121); *P* = .06	0.005 (–0.035 to 0.045); *P* = .79	–0.020 (–0.063 to 0.023); *P* = .37	–0.060 (–0.126 to 0.005); *P* = .07	0.001 (–0.015 to 0.018); *P* = .87
Oncologic	0.047 (0.016 to 0.078); *P* = .003	–0.008 (–0.032 to 0.016); *P* = .52	0.015 (–0.026 to 0.055); *P* = .47	0.055 (0.016 to 0.095); *P* = .006	0.009 (–0.019 to 0.038); *P* = .52	0.040 (0.007 to 0.073); *P* = .02	0.015 (–0.008 to 0.038); *P* = .19
Neurologic	0.006 (–0.033 to 0.045); *P* = .77	–0.002 (–0.024 to 0.021); *P* = .89	0.035 (–0.006 to 0.076); *P* = .09	0.002 (–0.034 to 0.037); *P* = .92	–0.014 (–0.050 to 0.021); *P* = .43	0.040 (–0.002 to 0.082); *P* = .07	–0.001 (–0.024 to 0.022); *P* = .94
Neonatal intensive	0.004 (–0.011 to 0.019); *P* = .59	–0.001 (–0.027 to 0.024); *P* = .93	0.016 (–0.015 to 0.046); *P* = .32	–0.011 (–0.037 to 0.014); *P* = .38	–0.002 (–0.024 to 0.021); *P* = .90	–0.023 (–0.039 to –0.006); *P* = .007	0.004 (–0.016 to 0.023); *P* = .72

^a^ Displays the coefficient from the difference-in-differences estimate using ordinary least squares regression adjusted for control variables in Table 1 and with hospital and calendar year fixed effects. Results account for staggered entry into the 340B program.

^b^ 95% CIs are calculated using standard errors clustered at the hospital level.

^c^ Thirty-one hospitals were missing Social Vulnerability Index data.

## Discussion

We found that public hospitals newly participating in the 340B program from 2012 to 2018 were more likely to expand unprofitable service line offerings compared with public hospitals that never participated. However, service provisions at nonprofit hospitals were largely unaffected by the 340B program, except for an increase in oncologic service offerings. Public hospitals were associated with a marginally significant increase in both substance use and inpatient psychiatric services. These findings are notable given concerns regarding service closure across the US,^22,33^ and the increasing trends in substance use disorders and mental health conditions.^34,35^

These findings suggest that 340B participation may enable public hospitals to sustain unprofitable, yet essential services. This finding is concordant with the underlying mission of the 340B program to subsidize comprehensive services for patients who need safety net services. We interpret these findings as reflecting cross-subsidization of unprofitable service lines indicating that the financial benefits of 340B participation were used to support access, regardless of the extent to which a service line received 340B savings. This is consistent with prior evidence suggesting that hospitals reduced the provision of psychiatric and substance use services in association with a decline in the profitability of cardiac services.^36^ In this case, 340B participation likely lowered the pharmaceutical cost of services that were reliant on hospital-based therapeutics, and the financial benefits were then used to sustain unprofitable service lines at public hospitals.

Although we found that the 340B program was associated with an increase in services at public hospitals, there was no meaningful change in unprofitable service provisions at nonprofit hospitals. This evidence is consistent with several past studies that found no association between hospital 340B program participation and improved access to safety net services.^7,8,10^ The finding persisted when stratifying our sample by geographic area, SVI, and critical access hospital designation, indicating that nonprofit hospitals located in socially vulnerable areas were not more likely to provide unprofitable services after 340B participation despite a possibly high need. Given that the US is reliant on hospitals to provide safety net services to patients who have low income and are uninsured,^37^ sustaining access to services is needed to improve population health and reduce disparities in outcomes.

### Limitations

First, there are important differences between hospitals that qualify for the 340B program and those that do not. Although we attempted to control for several hospital and market-level confounders, selection bias may remain. The comparison group included hospitals that do not qualify for 340B participation due either to payer mix or unmeasured management quality. There is also evidence that some hospitals manipulate their Disproportionate Share Hospital percentage to become eligible for the 340B program.^38^ Furthermore, the number of public hospitals in the sample is limited and the analyses may have been underpowered in some cases.

## Conclusions

The findings of this cohort study indicate that the US 340B Drug Pricing Program was associated with an increase in unprofitable service lines among public hospitals, but not among nonprofit hospitals. These findings suggest that the 340B program subsidies translated into increased access to unprofitable services at some, but not all, hospitals that serve disproportionate numbers of patients who require safety net services.

Increased regulatory oversight and program transparency may help to hold nonprofit hospitals accountable for using 340B program savings to sustain or expand comprehensive service offerings. Likewise, it is important to consider that increased regulation or compliance complexity may deter smaller and/or low-resourced hospitals from participating in the program due to the cost of meeting program compliance requirements. Policy discussion surrounding eligibility for the 340B program should recognize the heterogeneity in how program savings are used and support hospitals that are able to improve access to care for the patients they serve.

## Notes

Supplement 1. eMethods 1. American Hospital Association (AHA) Survey Services Definitions eMethods 2. Centers for Disease Control and Prevention Social Vulnerability Index Measure eMethods 3. Model Specification eTable 1. Baseline Hospital and Market Characteristics by New vs Always 340B Participation and Hospital Ownership eTable 2. Difference-in-Differences Estimates of Service Provisions Using a Balanced Panel eTable 3. Difference-in-Differences Estimates Using Logistic Regression eTable 4. Difference-in-Differences Estimates by Whether Hospital Offered Service at Start of the Study Period eReferences

Supplement 2. Data Sharing Statement
