# Supplementary material for: US Hospital Service Availability and New 340B Program Participation
Source: JAMA Health Forum. 2024 May 3;5(5):e240833. doi: 10.1001/jamahealthforum.2024.0833 (PMC11069079; doi:10.1001/jamahealthforum.2024.0833)
Supplement: Supplement 1. — eMethods 1. American Hospital Association (AHA) Survey Services Definitions eMethods 2. Centers for Disease Control and Prevention Social Vulnerability Index Measure eMethods 3. Model Specification eTable 1. Baseline Hospital and Market Characteristics by New vs Always 340B Participation and Hospital Ownership eTable 2. Difference-in-Differences Estimates of Service Provisions Using a Balanced Panel eTable 3. Difference-in-Differences Estimates Using Logistic Regression eTable 4. Difference-in-Differences Estimates by Whether Hospital Offered Service at Start of the Study Period eReferences [file jamahealthforum-e240833-s001.pdf]

Supplemental Online Content

Owsley KM, Hasnain-Wynia R, Rooks RN, Tung GJ, Mays GP, Lindrooth RC. US Hospital Service Availability and New 340B Program Participation. *JAMA Health Forum.* Published online May 3, 2024. doi:10.1001/jamahealthforum.2024.0833

eMethods 1. American Hospital Association (AHA) Survey Services Definitions

eMethods 2. Centers for Disease Control and Prevention Social Vulnerability Index Measure

eMethods 3. Model Specification

eTable 1. Baseline Hospital and Market Characteristics by New vs Always 340B Participation and Hospital Ownership

eTable 2. Difference-in-Differences Estimates of Service Provisions Using a Balanced Panel

eTable 3. Difference-in-Differences Estimates Using Logistic Regression

eTable 4. Difference-in-Differences Estimates by Whether Hospital Offered Service at Start of the Study Period

eReferences

This supplemental material has been provided by the authors to give readers additional information about their work.

© 2024 Owsley KM et al. *JAMA Health Forum.*

eMethods 1. American Hospital Association (AHA) Survey Services Definitions

**Measure**	**Notes**
Substance use care	Provides diagnostic and therapeutic services to patients with a medical illness characterized by clinically significant impairments in health, social function, and voluntary control over use of substances such as alcohol, prescription and non-prescription drugs. Substance use disorders range in severity, duration and complexity from mild to severe. Includes care for inpatient/residential treatment for patients whose course of treatment involves more intensive care than provided in an outpatient setting or where patient requires supervised withdrawal.
Burn care	Provides care to severely burned patients. Severely burned patients are those with any of the following: (1) second-degree burns of more than 25% total body surface area for adults or 20% total body surface area for children: (2) third-degree burns of more than 10% total body surface area; (3) any severe burns of the hands, face, eyes, ears, or feet; or (4) all inhalation injuries, electrical burns, complicated burn injuries involving fractures and other major traumas, and all other poor risk factors.
Inpatient psychiatric care	Provides acute or long-term care to patients with mental or emotional disorders, including patients admitted for diagnosis and those admitted for treatment of psychiatric disorders, on the basis of physicians' orders and approved nursing care plans. Long-term care may include intensive supervision to persons with chronic/severe mental illness.
Outpatient psychiatric	Services provided by the hospital that offer immediate initial evaluation and treatment to patients with mental or emotional disorders. Provides medical care, including diagnosis and treatment, of psychiatric outpatients.
Obstetrics	Services owned or provided by the hospital that is designated as either: (1) unit provides services for uncomplicated maternity and newborn cases; (2) unit provides services for uncomplicated cases, the majority of complicated problems, and special neonatal services; and (3) unit provides services for all serious illnesses and abnormalities and is supervised by a full-time maternal/fetal specialist.
Adult Cardiac Surgery	Services which include the diagnosis and treatment of diseases and disorders involving the heart and circulatory system. Includes minimally invasive procedures that include surgery done with only a small incision or no incision at all, such as through a laparoscope or an endoscope and more invasive major surgical procedures that include open chest and open heart surgery.
Orthopedic	Services provided for the prevention or correction of injuries or disorders of the skeletal system and associated muscles.
Oncology	Inpatient and outpatient services for patients with cancer, including comprehensive care, support and guidance in addition to patient

© 2024 Owsley KM et al. *JAMA Health Forum.*

	education and prevention, chemotherapy, counseling, and other treatment methods.
Neurological	Services provided by the hospital dealing with the operative and nonoperative management of disorders of the central, peripheral, and autonomic nervous systems.
Neonatal Intensive Care	A unit that must be separate from the newborn nursery providing intensive care to all sick infants including those with the very lowest birth weights (less than 1500 grams). NICU has potential for providing mechanical ventilation, neonatal surgery, and special care for the sickest infants born in the hospital or transferred from another institution. A full-time neonatologist serves as director of the NICU.

© 2024 Owsley KM et al. *JAMA Health Forum.*

eMethods 2. Centers for Disease Control and Prevention Social Vulnerability Index Measure

Analyses included a categorical variable for community-level social vulnerability, a significant factor in poor health and well-being outcomes.[1-3] The Centers for Disease Control and Prevention Social Vulnerability Index (SVI) was used to measure community social vulnerability. We obtained this variable using data from the Agency for Healthcare Research and Quality Social Determinants of Health Database.[4] SVI is constructed using fifteen social factors to rank counties by four distinct categories: socioeconomic status (below poverty, unemployment, income, no high school diploma); household composition and disability (aged 65 or older, aged 17 or younger, disability, single-parent household); minority status and language (racial or ethnic minority group, English language comprehension); and transportation and housing type (multi-unit housing structure, mobile home, crowded housing, vehicle access, group quarters).[5] Each census tract is ranked based on percentiles ranging from 0 to 1 for the four subthemes and overall vulnerability index, with higher values indicating greater vulnerability. We linked county-level SVI to the hospital dataset using county FIPS codes. We then categorized hospitals into SVI terciles (low, medium, and high SVI).

eMethods 3. Model Specification

In all analyses, we use the Callaway and Sant'Anna approach to adjust for the staggered treatment initiation of 340B participation using the CSDID STATA package.[6] This approach estimates all 2X2 difference-in-differences parameters for each treatment-timing group to obtain a "cohort-time average treatment effect", allowing for heterogeneity across treatment cohort groups.[7] We present this aggregated treatment effect for all analyses except the event study and the logit specification (eTable 3). Our specification includes hospital and time fixed effects. Standard errors were clustered at the hospital-level. The primary specification is as follows:

$$SERV_{ht} = \alpha_h + \beta_1 New340B_{ht} + \delta_t + X_{ht} + \varepsilon_{ht} \qquad (1)$$

where 'h' denotes hospital and 't' denotes calendar year. $SERV_{ht}$ is service availability for hospital 'h' in calendar year 't' , α_h is a hospital fixed effect, $New340B_{ht}$ is equal to one if hospital 'h' participates in the 340B program in calendar year 't'. The vector of control variables, X_{ht}, includes time-varying hospital and market-level controls listed in Table 1 of the manuscript and δ_t is a calendar year fixed effect. The coefficient, β_1, measures the average change in service availability relative to never participating hospitals.

We also estimated an event study specification to assess the trends before and after an acquisition. For the event study results, the approach estimates the dynamic treatment effects across each year relative to 340B participation, across all cohorts. We used the 'LONG2' option in the CSDID package to calculate dynamic treatment effect estimates using the year before participation (t-1) as a reference period. The specification is identical to Equation 1 except the model includes leads and lags of treatment to indicate the number of years a hospital is from the 340B participation year:

$$SERV_{ht} = \alpha_h + \sum_{j=0}^{5} \beta_j (Lag\ j)_{ht} + \sum_{k=1}^{5} \gamma_k (Lead\ k)_{ht} + \delta_t + X_{ht} + \varepsilon_{ht} \qquad (2)$$

.

© 2024 Owsley KM et al. *JAMA Health Forum.*

eTable 1. Baseline Hospital and Market Characteristics by New vs Always 340B Participation and Hospital Ownership

	No. (%) or Mean (SD)					
	Not-for-profit Hospitals			Public Hospitals		
	New 340B Hospitals from 2012-2018 (n=762)	Always 340B Hospitals Before 2012 (n=851)	*P* value	New 340B Hospitals from 2012-2018 (n=312)	Always 340B Hospitals Before 2012 (n=309)	*P* value
Hospital Characteristics						
No. of Admissions						
<1000	198 (26.0%)	216 (25.4%)	.033	185 (59.3%)	155 (50.2%)	.04
1000-9999	300 (39.4%)	385 (45.2%)		85 (27.2%)	113 (36.6%)	
10,000+	264 (34.6%)	250 (29.4%)		42 (13.5%)	41 (13.3%)	
Critical Access Designation	252 (33.1%)	354 (41.6%)	<.001	199 (63.8%)	188 (60.8%)	.45
Multi-hospital System	540 (70.9%)	620 (72.9%)	.375	108 (34.6%)	101 (32.7%)	.61
Teaching Hospital	72 (9.4%)	82 (9.6%)	.899	25 (8.0%)	14 (4.5%)	.07
Top Quartile Medicaid share	343 (45.0%)	382 (44.9%)	.960	122 (39.1%)	123 (39.8%)	.86
Top Quartile Medicare share	187 (24.5%)	204 (24.0%)	.790	155 (49.7%)	120 (38.8%)	.007
Case Mix Index, mean (SD)	1.483 (0.214)	1.474 (0.284)	.484	1.457 (0.208)	1.333 (0.288)	<.001
Market Characteristics						
Herfindahl-Hirschman Index, mean (SD)	0.186 (0.148)	0.179 (0.137)	.330	0.184 (0.137)	0.192 (0.144)	.52
For-profit Market Share	0.086 (0.124)	0.080 (0.114)	.349	0.161 (0.158)	0.136 (0.148)	.04
Social Vulnerability Index						
Bottom tercile	203 (26.6%)	266 (31.3%)	.063	90 (28.8%)	96 (31.1%)	.58
Middle tercile	254 (33.3%)	286 (33.6%)		90 (28.8%)	95 (30.7%)	
Top tercile	305 (40.0%)	299 (35.1%)		132 (42.3%)	118 (38.2%)	
Median Income ($1,000), mean (SD)	46.4 (10.53)	44.3 (9.8)	<.001	44.3 (9.7)	42.5 (8.7)	.01
Percent Uninsured, mean (SD)	16.6 (5.5)	17.0 (5.0)	.114	19.4 (5.6)	19.2 (5.6)	.76
Percent White, mean (SD)	80.8 (17.1)	81.9 (16.7)	.159	82.6 (16.5)	81.8 (16.3)	.51
Percent Above Age 65, mean (SD)	14.8 (3.9)	14.7 (3.6)	.435	15.7 (4.6)	15.4 (3.9)	.48
Drug Death Rate, mean (SD)	15.0 (6.7)	14.7 (7.4)	.456	16.2 (6.9)	15.8 (7.3)	.47
Rural	389 (51.0%)	523 (61.5%)	<.001	146 (46.8%)	134 (43.4%)	.03

© 2024 Owsley KM et al. *JAMA Health Forum.*

State expanded Medicaid	542 (71.1%)	610 (71.7%)	.807	166 (53.2%)	175 (56.6%)	.39

Notes: The statistics represent either the mean (standard deviation) or number (percent). Hospitals' first observation year in the dataset was used for New 340B hospitals. Always 340B hospital observations were in 2010. New 340B hospitals represent hospitals that began participating in the 340B program from 2012-18. Always 340B participating hospitals represent those that participated in the before 2012. The p-value indicates the difference between new and always 340B hospitals using student t-tests for continuous and chi-squared tests for binary variables.

© 2024 Owsley KM et al. *JAMA Health Forum.*

eTable 2. Difference-in-Differences Estimates of Service Provisions Using a Balanced Panel

	Difference-in-Differences Estimate[a] (95% CI)[b]		
		By Hospital Ownership	
	Overall (n=1,157)[c] (1)	Not-for-Profit (n=990)[c] (2)	Public (n=167)[c] (3)
**Total Unprofitable Services**	0.049	0.019	0.162
	(–0.014 - 0.111)	(–0.044 - 0.082)	(–0.019 - 0.343)
	P=.13	*P*=.56	*P*=.08
**Any Unprofitable Services**	0.008	0.011	−0.016
	(–0.013 - 0.030)	(–0.009 - 0.031)	(–0.086 - 0.054)
	P=.45	*P*=.28	*P*=.65
Substance Use	0.013	0.004	0.039
	(–0.011 - 0.038)	(–0.021 - 0.030)	(–0.025 - 0.102)
	P=.28	*P*=.73	*P*=.24
Inpatient Psychiatric	0.003	–0.014	0.032
	(–0.024 - 0.029)	(–0.039 - 0.012)	(–0.057 - 0.121)
	P=.84	*P*=.29	*P*=.48
Outpatient Psychiatric	0.023	0.018	0.091
	(–0.012 - 0.058)	(–0.018 - 0.055)	(–0.026 - 0.207)
	P=.20	*P*=.32	*P*=.13
Burn Care	–0.004	–0.007	0.011
	(–0.018 - 0.011)	(–0.023 - 0.008)	(–0.032 - 0.055)
	P=.62	*P*=.34	*P*=.61
Obstetrics	0.015	0.018	0.016
	(–0.002 - 0.032)	(–0.002 - 0.038)	(–0.038 - 0.069)
	P=.08	*P*=.07	*P*=.56
**Total Profitable Services**	0.017	0.035	–0.141
	(–0.039 - 0.073)	(–0.023 - 0.093)	(–0.357 - 0.074)
	P=.54	*P*=.24	*P*=.20
**Any Profitable Services**	–0.001	0.011	–0.114
	(–0.015 - 0.013)	(0.001 - 0.020)	(–0.211 - –0.016)
	P=.88	*P*=.03	*P*=.02
Cardiac Surgery	–0.012	–0.014	–0.008
	(–0.025 - 0.002)	(–0.029 - 0.001)	(–0.064 - 0.048)
	P=.10	*P*=.06	*P*=.79
Orthopedic	–0.001	0.011	–0.075
	(–0.020 - 0.017)	(–0.007 - 0.029)	(–0.169 - 0.018)
	P=.89	*P*=.24	*P*=.12

© 2024 Owsley KM et al. *JAMA Health Forum.*

Oncology	0.018	0.029	–0.027
	(–0.008 - 0.044)	(0.002 - 0.056)	(–0.117 - 0.063)
	P=.17	*P*=.04	*P*=.56
Neurological	0.002	0.006	–0.026
	(–0.024 - 0.027)	(–0.022 - 0.034)	(–0.109 - 0.057)
	P=.90	*P*=.68	*P*=.55
Neonatal Intensive	0.005	0.002	–0.058
	(–0.017 - 0.027)	(–0.020 - 0.024)	(–0.185 - 0.069)
	P=.67	*P*=.87	*P*=.37

a. Displays the coefficient from the difference-in-differences estimate using ordinary least squares regression adjusted for control variables in Table 1 and with hospital and calendar year fixed effects. Results account for staggered entry into the 340B program. The sample comprises non-critical access hospitals.
b. 95% Confidence intervals are calculated using standard errors clustered at the hospital level.
c. Denotes number of hospitals in the sample.

© 2024 Owsley KM et al. *JAMA Health Forum.*

eTable 3. Difference-in-Differences Estimates Using Logistic Regression

	Difference-in-Differences Estimate[a] (95% CI)[b]		
		By Hospital Ownership	
	Overall (n=1,630)[c] (1)	Not-for-Profit (n=1,387)[c] (2)	Public (n=243)[c] (3)
**Substance Use**	0.021	0.009	0.086
	(–0.005 – 0.046)	(–0.020 - 0.038)	(0.033 - 0.138)
	P=.116	*P*=.54	*P*=.001
**Inpatient Psychiatric**	0.037	0.026	0.092
	(0.003 - 0.072)	(–0.013 - 0.064)	(0.009 - 0.175)
	P=.04	*P*=.19	*P*=.03
**Outpatient Psychiatric**	0.023	0.025	0.003
	(–0.011 - 0.058)	(–0.013 - 0.064)	(–0.082 - 0.088)
	P=.18	*P*=.20	*P*=.95
**Burn Care**	0.009	0.002	0.027
	(–0.011 - 0.028)	(–0.018 - 0.022)	(–0.029 - 0.083)
	P=.39	*P*=.87	*P*=.35
**Obstetrics**	0.024	0.022	0.023
	(0.004 - 0.044)	(0.000 - 0.044)	(–0.026 - 0.072)
	P=.02	*P*=.05	*P*=.36
**Cardiac Surgery**	–0.003	0.004	–0.038
	(–0.026 - 0.019)	(–0.021 - 0.028)	(–0.100 - 0.024)
	P=.77	*P*=.76	*P*=.23
**Orthopedic**	–0.004	–0.004	–0.009
	(–0.018 - 0.011)	(–0.020 - 0.012)	(–0.055 - 0.037)
	P=.63	*P*=.61	*P*=.71
**Oncology**	0.026	0.036	–0.011
	(0.002 - 0.050)	(0.009 - 0.063)	(–0.071 - 0.050)
	P=.04	*P*=.008	*P*=.73
**Neurological**	0.001	0.000	–0.015
	(–0.024 - 0.026)	(–0.027 - 0.027)	(–0.074 - 0.044)
	P=.92	*P*=.99	*P*=.62
**Neonatal Intensive**	0.051	0.057	0.013
	(0.022 - 0.081)	(0.024 - 0.089)	(–0.077 - 0.103)
	P=.001	*P*=.001	*P*=.78

a. Displays the marginal effect from the difference-in-differences estimate using logistic regression adjusted for control variables in Table 1 and calendar year fixed effects.
b. 95% Confidence intervals are calculated using standard errors clustered at the hospital level.
c. Denotes number of hospitals in the sample.

© 2024 Owsley KM et al. *JAMA Health Forum.*

eTable 4. Difference-in-Differences Estimates by Whether Hospital Offered Service at Start of the Study Period

	Difference-in-Differences Estimate[a] (95% CI) [b]					
	Did Not Already Offer Service			Already Offered Service		
	Overall (1)	Not-for-Profit (2)	Public (3)	Overall (4)	Not-for-Profit (5)	Public (6)
Substance Use	–0.002	–0.008	0.022	0.123	0.100	0.590[c]
	(–0.019 - 0.014)	(–0.026 - 0.010)	(–0.033 - 0.076)	(–0.009 - 0.254)	(–0.029 - 0.230)	(–0.073 - 1.252)
	P=.78	*P*=.38	*P*=.43	*P*=.07	*P*=.13	*P*=.08
Inpatient Psychiatric	–0.004	–0.026	0.113	0.011	–0.002	–0.111[c]
	(–0.037 - 0.029)	(–0.060 - 0.007)	(0.022 - 0.204)	(–0.020 - 0.043)	(–0.033 - 0.028)	(–0.326 - 0.103)
	P=.82	*P*=.13	*P*=.02	*P*=.49	*P*=.89	*P*=.31
Outpatient Psychiatric	0.000	0.010	–0.008	0.064	0.041	0.927[c]
	(–0.035 - 0.035)	(–0.026 - 0.047)	(–0.106 - 0.090)	(0.007 - 0.121)	(–0.018 - 0.101)	(0.366 - 1.487)
	P=.99	*P*=.58	*P*=.88	*P*=.03	*P*=.17	*P*=.001
Burn Care	–0.005	–0.008	0.024	–0.609	0.350[c]	–0.066[c]
	(–0.015 - 0.005)	(–0.019 - 0.004)	(–0.011 - 0.059)	(-2.014 - 0.797)	(–0.110 - 0.809)	(–0.207 - 0.075)
	P=.31	*P*=.19	*P*=.18	*P*=.40	*P*=.14	*P*=.36
Obstetrics	–0.030	–0.037	0.000[c]	0.027	0.022	0.056
	(–0.100 - 0.039)	(–0.097 - 0.023)	(–0.462 - 0.462)	(0.008 - 0.045)	(0.002 - 0.041)	(0.007 - 0.105)
	P=.39	*P*=.23	*P*=.99	*P*=.004	*P*=.03	*P*=.03
Cardiac Surgery	–0.019	–0.025	–0.007	–0.005	–0.004	0.113[c]
	(–0.037 - –0.000)	(–0.046 - –0.004)	(–0.078 - 0.063)	(–0.032 - 0.021)	(–0.031 - 0.022)	(–0.494 - 0.721)
	P=.05	*P*=.02	*P*=.84	*P*=.70	*P*=.74	*P*=.71
Orthopedic	0.117	0.236	–0.283	–0.006	–0.004	–0.016[c]
	(–0.052 - 0.285)	(–0.023 - 0.495)	(–0.567 - 0.001)	(–0.018 - 0.005)	(–0.016 - 0.008)	(–0.075 - 0.043)
	P=.17	*P*=.07	*P*=.05	*P*=.30	*P*=.51	*P*=.60
Oncology	0.081	0.101	0.061	0.000	0.008	0.002
	(0.004 - 0.159)	(0.007 - 0.196)	(–0.097 - 0.220)	(–0.019 - 0.019)	(–0.014 - 0.029)	(–0.069 - 0.074)
	P=.04	*P*=.04	*P*=.45	*P*=.99	*P*=.47	*P*=.96

© 2024 Owsley KM et al. *JAMA Health Forum.*

Neurological	0.005	–0.001	0.007	–0.006	–0.003	0.030
	(–0.060 - 0.070)	(–0.087 - 0.085)	(–0.108 - 0.122)	(–0.027 - 0.014)	(–0.026 - 0.019)	(–0.048 - 0.108)
	P=.89	*P*=.98	*P*=.90	*P*=.55	*P*=.78	*P*=.45
Neonatal Intensive	0.015	0.010	–0.010	–0.012	–0.025	0.906[c]
	(–0.009 - 0.039)	(–0.016 - 0.036)	(–0.103 - 0.083)	(–0.050 - 0.027)	(–0.063 - 0.013)	(0.110 - 1.702)
	P=.23	*P*=.45	*P*=.84	*P*=.55	*P*=.20	*P*=.03

a. Displays the coefficient from the difference-in-differences estimate using ordinary least squares regression adjusted for control variables in Table 1 and with hospital and calendar year fixed effects. Results account for staggered entry into the 340B program. The sample comprises non-critical access hospitals.

b. 95% Confidence intervals are calculated using standard errors clustered at the hospital level.

c. Sample size is less than 100 hospitals.

© 2024 Owsley KM et al. *JAMA Health Forum.*

References

1. Paul R, Arif A, Pokhrel K, Ghosh S. The association of social determinants of health with COVID-19 mortality in rural and urban counties. *J Rural Health*. 2021;37(2):278-286.
2. Singh GK, Daus GP, Allender M, et al. Social determinants of health in the United States: addressing major health inequality trends for the nation, 1935-2016. *International Journal of MCH and AIDS*. 2017;6(2):139.
3. Hardy RY, Liu GC, Kelleher K. Contribution of social determinant of health factors to rural-urban preventive care differences among medicaid enrollees. *Academic Pediatrics*. 2021;21(1):93-100.
4. Agency for Healthcare Research and Quality. SDOH Database (v1) from Centers for Disease Control and Prevention Agency for Toxic Substances and Disease Registry Geospatial Research, Analysis, and Services Program. Social Vulnerability Index 2018, 2016, 2014, and 2010. Accessed November 17, 2022, https://www.atsdr.cdc.gov/placeandhealth/svi/data_documentation_download.html
5. Centers for Disease Control and Prevention. CDC SVI Documentation 2018. https://www.atsdr.cdc.gov/placeandhealth/svi/documentation/SVI_documentation_2018.html
6. Callaway B, Sant'Anna PH. Difference-in-differences with multiple time periods. *J Econom*. 2021;225(2):200-230.
7. Baker AC, Larcker DF, Wang CC. How much should we trust staggered difference-in-differences estimates? *Journal of Financial Economics*. 2022;144(2):370-395.

© 2024 Owsley KM et al. *JAMA Health Forum.*
