# Supplementary material for: US Hospital Service Availability and New 340B Program Participation
Source: JAMA Health Forum. 2024 May 3;5(5):e240833. doi: 10.1001/jamahealthforum.2024.0833 (PMC11069079; doi:10.1001/jamahealthforum.2024.0833)
Supplement: Supplement 2. — Data Sharing Statement [file jamahealthforum-e240833-s002.pdf]

Data Sharing Statement

Owsley. US Hospital Service Availability and New 340B Program Participation. *JAMA Health Forum*. Published May 03, 2024. doi:10.1001/jamahealthforum.2024.0833

Data
Data available: No

Additional Information
Explanation for why data not available: The American Hospital Association Survey data is proprietary and unavailable for public release.
